# Above-versus below-elbow casting for conservative treatment of distal radius fractures: a randomized controlled trial and study protocol

**DOI:** 10.1186/s12891-018-2007-9

**Published:** 2018-03-27

**Authors:** Aldo Okamura, Gabriel Maciel de Mendonça, Jorge Raduan Neto, Vinicius Ynoe de Moraes, Flavio Faloppa, João Carlos Belloti

**Affiliations:** 1 Hospital Municipal do Campo Limpo Dr. Fernando Mauro Pires da Rocha, Estrada de Itapecerica, 1661 - Campo Limpo, São Paulo, SP 05835-005 Brazil; 2 Hand, Arm and Shoulder Surgery Unit, Department of Orthopedics and Traumatology, Universidade Federal de São Paulo, UNIFESP/EPM, Rua Borges Lagoa, 778 Vila Clementino, São Paulo, SP Brazil

**Keywords:** Distal radius fracture, Conservative treatment, Treatment outcome, Randomized, Prospective

## Abstract

**Background:**

A variety of cast options are available for the non-surgical treatment of distal radius fractures (DRF) in adults. However, the literature is inconclusive regarding the need to immobilize the elbow joint after reduction in order to prevent rotation of the forearm in order to maintain the reduction of DRF. This study aimed to evaluate the best method of immobilization between above-elbow (AE) and below-elbow (BE) cast groups at the end of six-month follow-up.

**Methods:**

This is a randomized clinical trial with parallel groups and a blinded evaluator. There are two non-surgical interventions: AE and BE. Patients will be randomly assigned. A hundred twenty eight consecutive adult patients with acute (up to 7 days) displaced DRF of type A2, A3, C1, C2 or C3 by the Arbeitsgemeinschaft für Osteosynthesefragen (AO) classification will be included. The primary outcome will be the maintenance of reduction by evaluation of radiographic parameters and Disabilities of the Arm, Shoulder and Hand Questionnaire (DASH). Secondary outcomes include function measured by Patient Rated Wrist Evaluation (PRWE), pain measured by the Visual Analogue Scale (VAS), objective functional evaluation (goniometry and dynamometry) and rate of complications. Evaluations will be performed at 1, 2, 3, 4, 6, 8, 12 and 24 weeks. For the Student’s t-test, a difference of 10 points in DASH score, with 95% confidence interval, a statistical power of 95%, and 20% sampling error. We consider an extra 10% for balancing follow up losses results in 64 patients per group.

**Discussion:**

Results from this study protocol will help to define the need for elbow immobilization in maintenance of reduction, as well as functional performance of below elbow cast versus above elbow cast immobilization during the immobilization period.

**Trial registration:**

NCT03126175 (http://clinicaltrials.gov). April 24, 2017.

## Background

Although distal radius fractures (DRF) are among the most frequent of the upper limb [1], the best method of treatment and outcome of these fractures has not yet been fully defined [2, 3]. Regarding non-surgical treatment, Cochrane review based on randomized controlled trials has concluded there are controversial in terms of the type of casting to be applied after the initial fracture reduction and there is no conclusive evidence of difference in outcome between different positions and methods of plaster and brace management for the common types of DRF [4–6].

Below-elbow (BE) splinting is easier to apply, is lower in cost, lighter, provides greater comfort, better function for daily life activities and less articular stiffness of the elbow [7–9]. Casts that include the elbow joint, which prevents the rotation of the forearm, may result in greater stability of the fracture and less risk of loss of reduction and need for re-reduction [10–12]. Other studies found similar results between immobilization methods in maintaining the initial fracture reduction [13, 14].

This study is based on the hypothesis that above-elbow (AE) splint immobilization in patients with DRF will present better results for loss of reduction and radiographic parameters, but more complication rate and worse functional outcomes when compared to below-elbow (BE) immobilization methods at the end of a six-month follow-up.

## Methods/design

### Aim

To determine the best method of immobilization in patients with distal radius fractures at the end of a six-months: below-elbow versus above-elbow cast.

### Design and setting

Randomized controlled trial developed at Federal University of São Paulo - UNIFESP and Hospital Municipal Dr. Fernando Mauro Pires da Rocha - SP.

### Participant characteristics

**Fig. 1:**
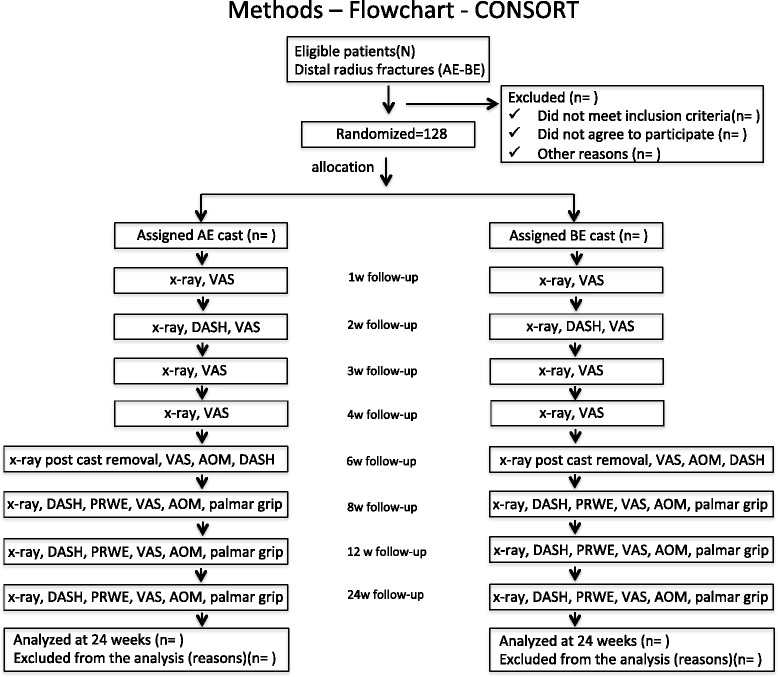
Flowchart of patients included in this study

Adults with growth plate closure, both genders, with unilateral and closed acute displaced DRF (up to 1 week), associated or not with the ulnar styloid fractures with no other fractures, which may be closed reduced and meet inclusion criteria (Fig. 1).

### Inclusion criteria

Displaced and reducible fractures classified by AO as type A2, A3, C1, C2 and C3 will be included if one of these conditions is present.

- Radial height – loss >2 mm [15–19].
- Radial Inclination - loss >4° [17, 20, 21].
- Dorsal angulation >10^o^ [5, 18, 20].
- Positive ulnar variance – loss >3 mm [19–21].
- Intra-articular step off or gap – >2 mm [5, 19, 22].
- Carpal malalignment [19, 23].

The contralateral side is used as a reference.

### Exclusion criteria

Patients presenting one or more of the following criteria will be excluded from this study:

- Open fractures, bilateral fracture or associated with tendon or neurovascular lesions.
- Associated carpal fractures.
- Marginal fractures or fractures from shearing mechanism.
- Fractures with palmar deviation (Smith’s fracture).
- Irreducible fractures (closed method).
- Prior history of a degenerative or traumatic disorder of the affected or contralateral wrist joint.
- Systemic diseases or traumatic lesions associated with fracture that restrict the application of methods or the evaluation of results.
- Cognitive deficit that does not allow the patient to understand the elements of the functional evaluation.
- Consent Form Refusal.

### Radiological measurements

The volar tilt, the radial inclination, the radial height, the ulnar variance and the intra-articular step off or gap were determined on posteroanterior (PA) and lateral (L) radiographs views obtained using a standardised procedure [24].

The standard method of obtaining a PA radiograph is with the shoulder in 90° of abduction, the elbow in 90° of flexion and the wrist in a neutral position. For the lateral view, the shoulder is adducted and the elbow is in 90° of flexion with the hand positioned in the same plane as the humerus [19].

The volar tilt, also called palmar tilt is measured on the lateral view and refers to the distance between a line through the dorsal and palmar boundary points of the radial joint surface and the perpendicular to the longitudinal axis of the radial shaft.

The radial inclination, also know as radial deviation is measured on the PA view and refers to the distance between a line through the radial and ulnar boundaries of the radial joint surface and the perpendicular to the longitudinal axis of the radial shaft.

The radial height, also called radial lenght is measured on the PA view and refers to the difference in axial direction of the radius between the distal tip of the radial styloid and the most distal aspect of the ulnar articular surface.

The ulnar variance, also called the radioulnar index is measured on the PA view and refers to the vertical distance between a line parallel to the medial corner of the articular surface of the radius and a line parallel to the most distal point of the articular surface of the ulnar head, both of which are perpendicular to the long axis of the radius.

The intra-articular step off or gap is measured on PA or lateral view and refers articular incongruity.

The carpal alignment is measured on lateral view. Two lines are drawn, one along the long axis of the capitate and other along the long axis of the radius. The lines do intersect within the carpus.

### Initial treatment

**Fig. 2:**
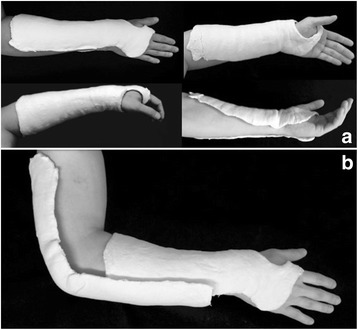
Types of immobilization. Below-elbow cast (**a**). Above-elbow cast (**b**)

All the patients with a distal radius fracture who arrive at the emergency room will undergo a standard protocol with clinical and radiographic examination (bilateral x-rays of the wrist in PA and lateral views). After applying the inclusion and exclusion criteria, eligible individuals will be informed about the nature and purpose of the study, by reading the “Consent Form” and after signing it they will be included. On a pre-scheduled date (up to 7 days), the study participant will be referred to the main operating room to be anesthezied before closed reduction of the fracture under radioscopy control. The reducibility criteria will be evaluated and patients that have reducible fracture will be randomized and treated by one of the two methods of the study (Fig. 2). Patients that do not have closed reducible fracture will be excluded from the study and will receive surgical treatment (open reduction and internal fixation) on a date to be scheduled.

### Anesthesia

Intravenous anesthesia will be performed by aseptic technique. A simple bolus injection with Propofol (infusion rate 180 mcg.kg^− 1^.min^− 1^) in combination with opioid (fentanyl 5–10 mcg.kg^− 1^) adjusted to the individual needs of each patient and repeated as many times as necessary according to the anesthesiologist’s criteria [25, 26].

### Method for closed reduction and immobilization

The patient will be submitted to the closed reduction of the fracture through a traction and counter-traction technique. Materials needed for application of the two splinting techniques will be available in the operating room. Initially, all patients will receive a short radial splint that will be performed with a 20 cm wide gypsum cut to fit the thumb (Fig. 2a). The splint will be applied to the radial aspect of the wrist covering the volar and dorsal portion of the radius to the elbow. The splint will be moulded with three point fixation as described by Charnley [27]. The three points will be defined after a metal pointer will be placed beside the limb to identify the site of fracture by using the image intensification. Patients randomized to the above-elbow splint will receive a complementation of immobilization with a 15 cm width splint on the ulnar aspect of the forearm that begins at the middle of the forearm and extends into the armpit. The elbow will be immobilized at 90 degrees, and in a neutral position to block pronosupination (Fig. 2b). Cotton tubular mesh, cotton stripes and crepe bandage will be used in both bindings. Regardless of the immobilization adopted, all wrists will be positioned with slight flexion and ulnar deviation. Patients will be encouraged to actively move their fingers and the ipsilateral shoulder.

Patients with above-elbow immobilization will remain for 4 weeks with the splint followed by 2 weeks of below-elbow immobilization. The immobilization will be removed after 6 weeks.

### Clinical outcomes

The self-reported functional evaluation DASH and PRWE, visual analogue pain scale (VAS), radiographic measures, objective functional evaluation will be performed by independent evaluators at intervals provided in Table 1. For the outcomes at 8,12 and 24 weeks the evaluators will be blinded to the patient assignment groups. The minimum clinical follow-up will be 24 weeks, with the following parameters being considered to evaluate the results:

**Table 1:** Outcomes and measurement time

	1 W	2 W	3 W	4 W	6 W	8 W	12 W	24 W
X rays	x	x	x	x	x	x	x	x
DASH		x			x	x	x	x
PRWE						x	x	x
VAS	x	x	x	x	x	x	x	x
AOM					x	x	x	x
Palmar Grip						x	x	x

### Primary outcomes

#### Radiographic parameters

Maintenance of reduction by evaluation wrist radiographs in PA and lateral x-rays at the following intervals: one, two, three, four, six, eight, twelve and twenty-four weeks after fracture reduction.

The radial height, radial inclination, volar tilt, ulnar variance, intra-articular step off or gap and carpal alignment will be used to determine maintenance of reduction at every follow-up visit. Measurements will be made on the radiographs with a marker, straight edge, and protractor by two researchers independently on different occasions.

We will consider maintenance of reduction if:

- loss of reduction ≤2 mm in radial height
- loss of reduction ≤4 degrees in radial inclination
- dorsal angulation ≤10^o^
- ≤ 2 mm intra-articular step off
- positive ulnar variance ≤3 mm
- any carpal malalignment.

The contralateral side is used as a reference.

#### Patient-reported functional outcomes

Functional status will be evaluated by means of DASH questionnaire (validated for the Portuguese language) at the following intervals: two, six, eight, twelve and twenty-four weeks after fracture reduction [28]. The DASH was developed as an instrument for patients with upper-extremity injuries. The survey contains 30 questions related to the function of the hand, wrist, elbow, and shoulder based on the conditions to do certain activities in the past week, so the evaluations refer only after the beginning of the immobilization.

### Secondary outcomes

Patient Rated Wrist Evaluation – PRWE; [29] Pain (VAS - Visual Analogue Pain Scale); [30, 31] Objective functional evaluation (goniometry and dynamometry); and rate of complications and failures.

The PRWE score (validated for the Portuguese language) will be obtained at eight, twelve and twenty-four weeks. The PRWE contains 15 items that are specific to determining the degree of musculoskeletal disability related to the wrist [29].

Pain in the wrist, elbow and shoulder will be measured separately in all visits at one, two, three, four, six, eight, twelve and twenty-four weeks after fracture reduction by the Visual Analogue Pain Scale (VAS). This is a unidimensional measure of pain intensity, which has been widely used in diverse adult populations [30]. Pain in VAS is a continuous scale comprised of a horizontal line of 10 cm (100 mm) in length, anchored by two verbal descriptors, one for each symptom extreme by “no pain” (score of 0) and “pain as bad as it could be” or “worst imaginable pain” (score of 100). Participants are asked to report pain intensity in the last 24 h. The respondent is asked to place a line perpendicular to the VAS line at the point that represents their pain intensity. Using a ruler, the score is determined by measuring the distance (mm) on the 100 mm line between the “no pain” anchor and the patient’s mark, providing a range of scores from 0 to 100 [31].

#### Objective functional evaluation

Arcs of motion will be measurement for the wrist, and a goniometer will be employed to measure wrist flexion, extension, ulnar deviation, radial deviation and pronosupination at the six, eight, twelve and twenty-four week follow up visit. The flexion–extension of the elbow will be measurement at six, eight, twelve and twenty-four week follow-up visit.

Palmar grip strength with a digital dynamometer (Jamar Plus - Hand Dynamometer), at the following moments of treatment evolution: eight, twelve and twenty-four week follow-up visit.

#### Complications

Any clinical situation requiring treatment (clinical or surgical procedure) not provided in the protocol will be considered as a complication. All complications will be recorded for further stratification into major and minor complications.

In cases where there is loss of reduction, patients will be informed and surgical treatment indicated.

#### Statistical methods

Descriptive data will be exposed as means or proportions followed by standard deviations or 95% confidence intervals. As a method to confirm the effectiveness of the randomization, baseline data will be compared in the two groups of comparison. To ensure the normal distribution of data, we will use visual analysis and Shapiro-wilk test.

For comparison between proportions, we will consider Pearson’s chi-square test. For continuous data, we will use Student T test. Intra-group comparison (1, 2, 3, 4, 6, 8, 12 and 24 weeks) will be analyzed by paired Student T test or Wilcoxon (if data is not normally distributed). We will consider as significant when alpha < 0,05. To analyze the occurrence of complication after treatment, we intend to perform survival analysis associated with Kaplan-Meier curves, if we find greater than 20% complication in any of the comparison groups. All statistical analysis will be performed following intention to treat principle. Statistical advisors will be blinded to the treatment groups as an effort to decrease bias.

#### Randomization and masking

Patients will be randomly assigned using randomization software (available at: http://www.randomizer.org). The allocation of patients in the AE or BE groups will be performed using opaque envelopes numbered on their outer face with consecutive numbers (concealment). Additionally, the envelope will be opened only in the operating room after verification of fracture reducibility and the procedure will be delegated to a person who is not directly connected to the study.

#### Sample size calculation

Based on data derived from one recent randomized clinical trial on the subject [32]. We considered as relevant differences on DASH scores (clinically relevant) when scores are greater than 10 points and standard deviation 15 points [33]. To detect this difference (Student T-test) and statistical power of 95% resulted in a 58 patient sample size per group. We considered an extra 10% for balancing follow up losses. Thus, our inclusion target will be 64 patients per group. We considered the test as bicaudal.

## Discussion

This publication presents a randomized clinical trial of the non-operative treatment of DRF. Casts may be applied either “above elbow” or “below elbow”, depending on the particular type of injury and physician preference. Often, the plaster may extend above the elbow to help provide additional stability and neutralize the extensive forces that can be generated by natural movements of the arm and forearm. Above-elbow immobilization is the conservative treatment used by most of the Brazilian orthopedic surgeons (74%) [34].

Short arm immobilization has been used by many orthopedic surgeons around the world, who claimed equally beneficial results [8, 13]. Hence, controversy still persists regarding the length of the immobilization for the treatment of DRF [4, 5].

The value of the study includes all participants will be reduced in the main operating room under general intravenous anesthesia and with the aid of radioscopy which will allow better control of the pain and maximum quality in the reduction. All reductions and immobilizations will be performed by a single researcher, specialist in hand surgery. The follow up during the immobilization period will be weekly, with radiographic documentation, which allows the early identification of the reduction loss. This is the only trial to apply DASH questionnaire at the beginning and end of immobilization period (2 and 6 weeks) to compare the groups. Pain in the wrist, elbow and shoulder will be measured separately in all visits to verify the influence of immobilization on the elbow and shoulder joints. Adults of all ages will be evaluated, it is known that the DRF in the elderly has different behavior and prognosis when compared to the young [35–38]. Randomization will equalize the distribution homogeneously between the groups, allowing the sample to be faithful to the population.

Our study has several strengths and limitations. Weekly assessments increase the chance of follow-up loss, however a strict control will be adopted. The study presents limitations because the database was constructed based on measurements of X-ray films calculated manually with goniometer and pen, which may imply in unmeasured tolerance limits. To minimize this, the measurements were performed by two senior researchers independently at different times. All patients in this study will be users of the public health system, many of them may have difficulty responding to self-reported questionnaires. A trained assistant will be available in these cases. Another important point to consider is the work compensation in some patients who want secondary gains, which can influence the information collected.

The results from this randomized clinical trial study are expected to be published in december of 2019. We hope that the study results will provide an answer as to which is the best conservative treatment method for DRF.

## Abbreviations

AE: Above-elbow
AO: Arbeitsgemeinschaft für osteosynthesefragen
BE: Below-elbow
DASH: Disabilities of the arm, shoulder and hand
DRF: Distal radius fractures
Kg: Kilograms
L: Lateral
mcg: Micrograms
min: minutes
PA: Posteroanterior
PRWE: Patient rated wrist evaluation
UNIFESP: Federal University of São Paulo
VAS: Visual analogue scale
